# Development of acetabular anteversion in children with normal hips and those with developmental dysplasia of the hip: a cross-sectional study using magnetic resonance imaging

**DOI:** 10.1080/17453674.2020.1866928

**Published:** 2021-01-08

**Authors:** Wei Lu, Lianyong Li, Lijun Zhang, Qiwei Li, Enbo Wang

**Affiliations:** Department of Pediatric Orthopedics, Shengjing Hospital of China Medical University, Shenyang, Liaoning Province, China

## Abstract

Background and purpose — Acetabular anteversion (AA) is related to hip function. Most previous studies were based on radiographic investigations that determine osseous acetabular anteversion (OAA). But children’s acetabulum is mostly composed of cartilage; the cartilaginous acetabular anteversion (CAA) represents the real anteversion of the acetabulum. We measured OAA and CAA in children of various ages using MRI, and compared the developmental patterns between children with normal hips and those with developmental dysplasia of the hip (DDH).

Patients and methods — The OAA and CAA were measured on MRI cross-sections of the hips in 293 children with normal hips (average age 8 years), and in 196 children with DDH (average age 34 months). Developmental patterns of OAA and CAA in children with normal hips were determined through age-based cross-sectional analysis. Differences in OAA and CAA between children with normal hips and those with DDH were compared.

Results — Normal OAA increased from mean 8.7° (SD 3.2) to 12° (3.0) during the first 2 years of life and remained unchanged until 9 years of age. From 9 to 16 years, the OAA showed a minimal increase of 2°–3°. The normal CAA increased rapidly from a mean of 12° (3.1) to 15° (2.7) within the first 2 years of life, and remained constant at 15° (SD 3.4) until 16 years of age. The age-matched average OAA in the normal and DDH cases was 11° (3.2) and 15° (3.0), respectively (p < 0.001). The age-matched average CAA in normal and DDH cases was 17° (4.2) and 23° (4.5), respectively (p < 0.001). Similarly, there was a significant difference in OAA and CAA between the uninvolved hips in unilateral DDH and normal cases (p < 0.001).

Interpretation — The CAA was fully formed at birth in normal children, and remained unchanged until adulthood, whereas the OAA increased with age. The OAA and CAA were both over-anteverted in DDH children. MRI evaluation is of importance in children during skeletal development when planning hip surgery.

Acetabular anteversion (AA) is the direction of the acetabular opening relative to the axial planes. This is among the important quantitative indicators to describe acetabular tilt and is related to hip joint function. Proper AA facilitates the inclusion of the femoral head. The related position between the anterior and posterior walls of the acetabulum determines the normal angle of AA. At the same time, AA is also an important reference index for various pelvic rotational osteotomy or hip replacement procedures. Traditionally, AA is measured on 3D computed tomography (CT) (Li et al. 2009), which represents the osseous acetabular anteversion (OAA). However, children’s acetabulum is mostly composed of cartilage; the cartilaginous acetabular anteversion (CAA) determined by the cartilaginous anterior and posterior walls is the true AA, but cannot be measured by CT scan. The normal value of CAA in childhood is rarely reported.

Developmental dysplasia of the hip (DDH) includes delayed cartilage ossification or acetabulum margin dysplasia. Many previous studies have reported that over-anteversion of the acetabulum is one of the morphological abnormalities in children with DDH (Albinana et al. 2005, Jia et al. 2011). However, most previous studies were based on radiographic investigation, which therefore determined the OAA. The changes in CAA in DDH have never been established. Recognizing the true AA is an important aspect of the morphological evaluation of DDH and an important reference index for the surgical correction of acetabulum direction; thus, the evaluation of CAA is of clinical significance, especially in children during skeletal development.

Owing to the advantage of noninvasively distinguishing the osseous rim from the cartilaginous acetabulum in the immature hip, magnetic resonance imaging (MRI) has been widely used to evaluate the pathological abnormity of DDH (Mootha et al. 2010, Krasny et al. 1991, Falliner et al. 2002, Douira-Khomsi et al. 2010, Goronzy et al. 2019). In this retrospective cross-sectional MRI study we measured OAA and CAA in children of various ages using MRI, analyzed their developmental patterns, and compared the difference in OAA and CAA between children with normal hips and those with DDH.

## Patients and methods

### Samples and measurements

We performed a retrospective cross-sectional MRI study of children under 16 years of age who had underwent MRI (including the hip joint) from January 2008 to January 2018 in the authors’ institution. By using our Picture Archiving and Communication System (PACS), 2 pediatric orthopedics specialists (LYL and LJZ) read the pelvic MRI data. Patients with non-standard MRI examination or with other diseases of the hip such as DDH, femoral head necrosis, septic arthritis, or other syndromic disease of the hip were excluded. 293 consecutive children (586 hips) who underwent MRI examination because of non-neuromuscular and non-skeletal disease were included in the study. There were 147 boys and 146 girls with a mean age of 8.0 years (1 month to 16 years). All of the 586 hips were clinically normal, but with a diagnosis of lower limb pain, benign abdominal tumors, lymphangioma, or hemangioma in the cutaneous or subcutaneous tissues around the pelvis as the reason for the MRI examination.

Patients with DDH who were treated in Department of Pediatric Orthopedics of Shengjing Hospital from January 2008 to January 2018 were also included in the study. All patients were aged under 7 and had not been treated before. Patients with neuromuscular and syndromic DDH were excluded. 196 consecutive patients were reviewed in the study, including 159 girls and 37 boys (241 affected hips). Among these 196 children with DDH, 45 had bilateral involvement and 151 had unilateral involvement, including 95 and 56 patients with left and right hip involvement, respectively. The average age at reduction was 34 months (6–84 months). Patients with poor quality MRI scans and neuromuscular and pathological DDH were excluded. According to the International Hip Dysplasia Institute (IHDI) classification (Narayanan et al. 2015), 30 hips were classified as grade I, 33 hips as grade II, 65 hips as grade III, and 113 hips as grade IV.

All of the hips in children with normal hips and those with DDH were examined with standardized MR scans. The MR scans were performed using the 3.0 T Philips Medical System (Philips Achieva, Best, The Netherlands), including the pelvis and proximal femur with axial, sagittal, and coronal plane sequences. The patients were placed supine, with legs in a neutral position, and with a body array coil placed anterior and posterior to the pelvis. The MR scan was carried out under sedation before the MR examination in children aged < 4 years. The sequences included T1- and T2-weighted images obtained in the axial and coronal planes using 3-mm slice thickness and 0-mm interslice gap. All sequences used a TR of 4500 ms and TE of 120 ms in T2-weighted fast spin-echo; TR of 450 mms and TE of 12 ms in T1-weighted spin-echo; and matrix of 512 × 512.

**Figure 1.**
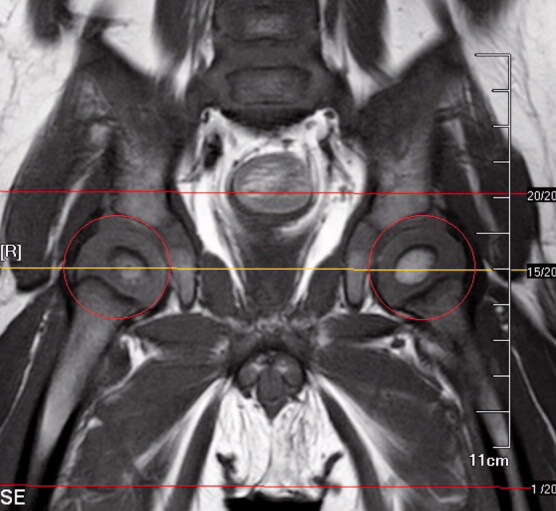
In the coronal position, the yellow line is the line passing through the center of the acetabulum. The corresponding axial section through the line is shown in Figure 2.

Using the MR scans, the OAA and CAA were measured by using the PACS. OAA and CAA were measured on the axial section of the hip located through the center of the acetabulum on the coronal section (Figure 1). On the axial section, line A was tangential to the most posterior points of both ischia. Line B was drawn perpendicular to line A. Line C was tangential to the outermost osseous anterior and posterior walls of the acetabulum. Line D was tangential to the outmost cartilaginous anterior and posterior walls of the acetabulum. The angle between lines B and C was defined as OAA, and that between lines B and D was defined as CAA (Figure 2).

Figure 2.Measurement of osseous acetabular anteversion (OAA) and cartilaginous acetabular anteversion (CAA). In the T1-weighted axial magnetic resonance image (MRI), line A is tangential to the most posterior points of both ischia. Line B is drawn perpendicular to line A.a. Line C is tangential to the outermost osseous anterior and posterior walls of the acetabulum and the angle between lines B and C is defined as the OAA.b. Line D is tangential to the outermost cartilaginous anterior and posterior walls of the acetabulum and the angle between lines B and D is defined as the CAA.

**Figure:**
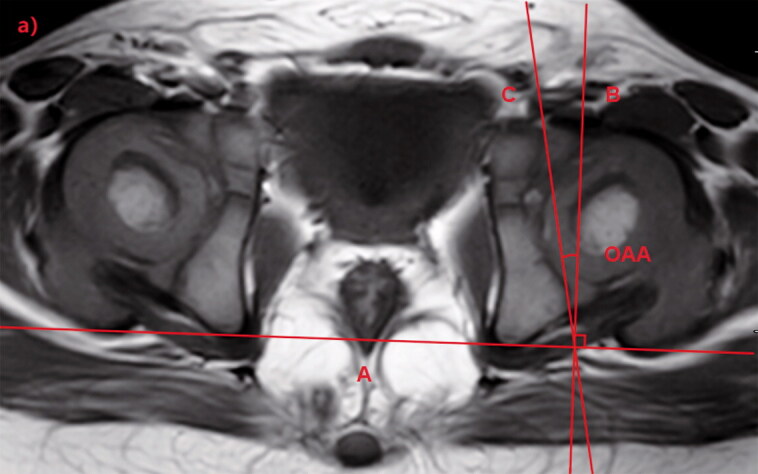

**Figure:**
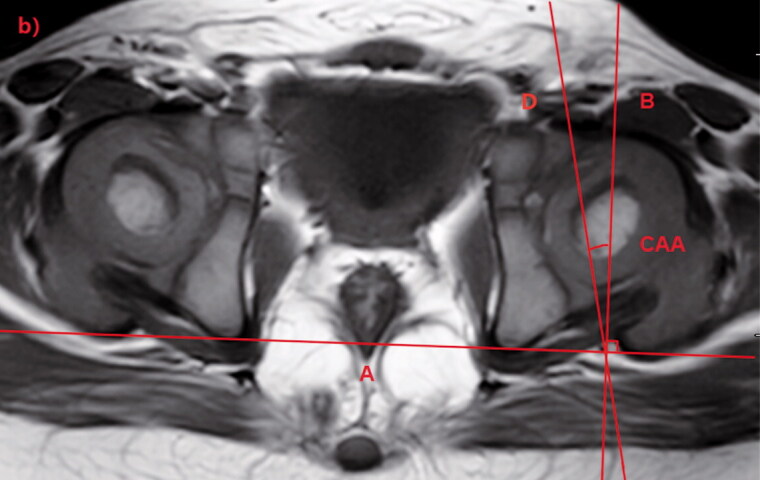

The OAA and CAA were measured (30 hips) 3 times independently, identified by initials only, to determine interobserver agreement; to evaluate intraobserver variation, the measurements were repeated 4 weeks later by initials.

### Statistics

Statistical analysis was performed using SPSS version 20.0 software (IBM Corp, Armonk, NY, USA). To evaluate the relationship between OAA and CAA, we used paired t-tests and Pearson correlation analysis. Student’s t-test was used to compare the difference in OAA and CAA between the normal and DDH cases. Differences in OAA and CAA among different age and IHDI classification groups were assessed with 1-way analysis of variance. A p-value < 0.05 was considered statistically significant.

Interobserver agreements between 3 sets of measurements of three observers and intraobserver agreement between 2 sets of measurements of an observer were analyzed using Pearson’s correlation coefficient and the intraclass correlation coefficient (ICC). According to the usual recommendations, the concordance was examined as follows: < 0.2 (bad), 0.2–0.4 (low), 0.4–0.6 (moderate), 0.6–0.8 (good), and > 0.8 (excellent).

### Ethics, funding, and potential conflicts of interest

This study was approved by the Institutional Ethical Committee. The parents or guardians of all study participants provided informed consent. The authors benefited from a funding program, the National Natural Science Foundation of China (81772296), and have no conflicts of interest to declare.

## Results

The intra- and interobserver agreements with 95% confidence intervals (CIs) were assessed by measuring the OAA and CAA repeatedly. For all measurements, the ICCs were > 0.8, indicating excellent agreement (Table 1).

**Table 1.** Assessment of intra- and interobserver agreements of osseous acetabular anteversion (OAA) and cartilaginous acetabular anteversion (CAA)

	OAA	CCA
Observers	ICC (95% CI)	ICC (95% CI)
WL–WL	0.997 (0.996–0.998)	0.976 (0.973–0.982)
WL–LYL	0.882 (0.858–0.911)	0.886 (0.864–0.911)
WL–LJZ	0.990 (0.987–0.992)	0.873 (0.849–0.892)
LYL–LJZ	0.898 (0.875–0.912)	0.901 (0.882–0.923)

ICC, intraclass correlation coefficient; CI, confidence interval.

P-value for each observer pair < 0.01

**Figure 3.**
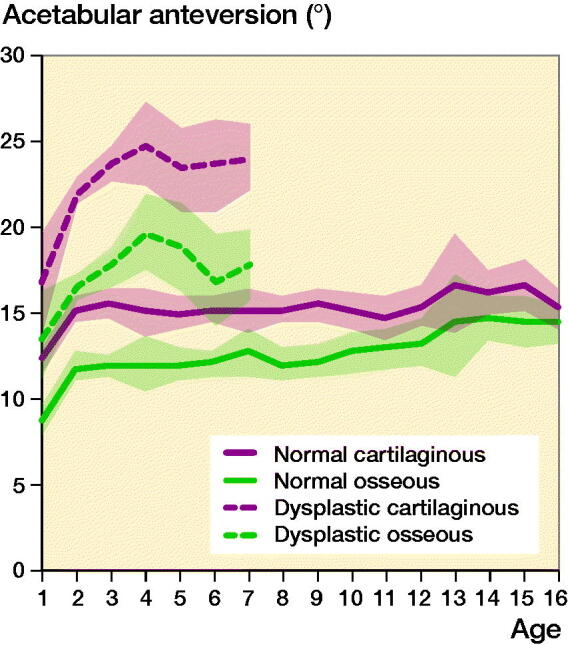
Mean trend lines with 95% CI of all measurements for osseous and cartilaginous acetabular anteversion versus age of children with normal hips and those with developmental dysplasia of the hip.

The mean OAAs and CAAs according to age in the 586 normal hips are summarized in Table 2. The normal OAA had a mean of 8.7° at 1 year of age, which increased progressively during the first 2 years of life, reaching a mean of 12° at 2 years of age. Then, it remained constant until the age of 9 years. At 9–16 years of age, it increased slightly by approximately 2° (Figure 3). Notably, the normal CAA had a different pattern of development from the OAA. Within the first 2 years of life, the CAA increased rapidly from a mean of 12° to 15°, and then stayed at a constant mean level of 15° until the age of 16 years (Figure 3). In addition, acetabular retroversion was found in none of the hips.

**Table 2.** Values of osseous (OAA) and cartilaginous acetabular anteversion (CAA) corresponding to age in normal children

	Hips	OAA (°)	CAA (°)
Age	n	Mean (SD) [95% Cl]	Mean (SD) [95% Cl]
1	58	8.7 (3.2) [7.9–9.5]	12.3 (3.1) [11.5–13.1]
2	48	11.8 (3.0) [11.0–12.7]	15.1 (2.7) [14.4–16.0]
3	40	12.0 (1.7) [11.4–12.5]	15.5 (2.4) [14.7–16.3]
4	16	12.0 (3.1) [10.4–13.7]	15.1 (2.6) [13.7–16.5]
5	36	11.9 (2.9) [11.0–12.9]	15.0 (2.7) [14.1–16.0]
6	44	12.1 (2.6) [11.3–12.8]	15.2 (2.6) [14.4–16.0]
7	28	12.7 (3.4) [11.4–14.0]	15.1 (3.2) [13.8–16.3]
8	48	12.0 (3.1) [11.1–12.9]	15.2 (2.8) [14.4–16.0]
9	36	12.2 (3.0) [11.2–13.2]	15.5 (3.0) [14.5–16.5]
10	38	12.7 (3.4) [11.6–13.8]	15.2 (3.1) [14.1–16.2]
11	32	12.9 (3.4) [11.7–14.1]	14.7 (3.3) [13.5–15.9]
12	40	13.3 (4.1) [11.9–14.6]	15.4 (3.6) [14.3–16.6]
13	20	14.4 (6.3) [11.4–17.3]	16.7 (6.2) [13.8–19.6]
14	30	14.7 (3.2) [13.5–15.9]	16.2 (3.2) [15.0–17.4]
15	34	14.5 (4.2) [13.0–15.9]	16.6 (4.3) [15.1–18.1]
16	38	14.4 (3.6) [13.2–15.5]	15.3 (3.7) [14.1–16.5]

CI, confidence interval; SD, standard deviation.

P-value for OAA versus CAA in each age group < 0.01

**Figure 4.**
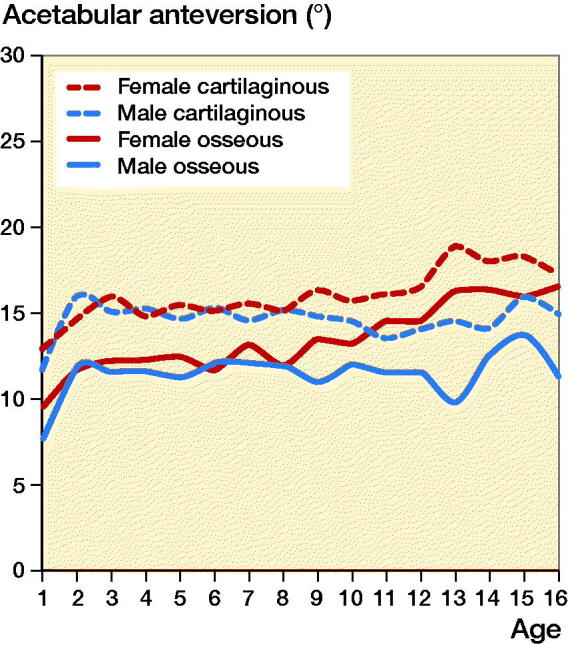
Osseous acetabular anteversion and cartilaginous acetabular anteversion between the sexes.

There was a positive correlation between OAA and CAA in the normal hips (r = 0.92, p < 0.001). For the OAA in the normal hips, there was a statistically significant difference between the girls and boys (Figure 4). From 0–9 years of age, the development trend of OAA between the sexes was basically the same. After the age of 9 years, OAA showed a more obvious increase in girls compared with boys until 16 years of age. The relationship of CAA between the sexes was similar to that of OAA (Figure 4).

The average OAA in normal and DDH cases was 11° and 15°, respectively (p < 0.001). The average CAA in normal and DDH cases was 17° and 23°, respectively (p < 0.001). Both OAA and CAA in children with DDH were larger than those in normal children, when the age of the individuals was matched (p < 0.001, Table 3, Figure 3). In DDH cases, the OAA in the uninvolved hip in unilateral DDH cases showed a mean of 14° and that of the age-matched children with normal hips showed a mean of 11° (p < 0.001). The CAA in the uninvolved hip in unilateral DDH cases showed a mean of 18°, and that of the age-matched children with normal hips showed a mean of 15° (p < 0.001), indicating that both OAA and CAA in the uninvolved hips were also over-anteverted.

**Table 3.** Values of osseous (OAA) and cartilaginous acetabular anteversion (CAA) corresponding to age in children with developmental dysplasia of the hip

	Hips	OAA (°)	CAA (°)
Age	n	Mean (SD) [95% Cl]	Mean (SD) [95% Cl]
1	13	13.7 (4.2) [11.2–16.3]	16.8 (5.0) [13.8–19.8]
2	121	16.6 (3.9) [15.9–17.3]	22.1 (4.3) [21.4–22.9]
3	50	17.8 (4.1) [16.6–19.0]	23.7 (3.7) [22.7–24.8]
4	19	19.8 (4.4) [17.7–22.0]	24.9 (5.1) [22.4–27.3]
5	14	18.9 (4.3) [16.4–21.4]	23.5 (4.2) [21.0–25.9]
6	14	17.0 (4.7) [14.3–19.7]	23.8 (4.6) [21.1–26.4]
7	10	17.9 (3.0) [15.8–20.0]	24.1 (2.5) [22.3–26.0]

CI, confidence interval; SD, standard deviation.

P-value for OAA versus CAA in each age group < 0.01

For the DDH cases, OAA was statistically significantly less than CAA, regardless of IHDI types (Table 4). OAA and CAA were similar among the IHDI types, except for the minimal difference (approximately 2°–3°) between type I and IV (p = 0.02), type II and IV (p < 0.001) for OAA; and between type III and IV (p = 0.01), type II and IV (p < 0.001) for CAA.

**Table 4.** Mean (SD) with 95% confidence intervals (CI) of osseous (OAA) and cartilaginous acetabular anteversion (CAA) in children with developmental dysplasia of the hip according to the IHDI classification

IHDI	Hips	OAA (°)	CAA (°)
types	n	Mean (SD) [95% Cl]	Mean (SD) [95% Cl]
I	30	16.0 (3.5) [14.7–17.4]	22.4 (4.2) [20.8–23.9]
II	33	15.3 (3.5) [14.1–16.6]	20.8 (4.5) [19.2–22.4]
III	65	17.0 (4.0) [16.0–17.9]	22.0 (4.5) [20.8–23.1]
IV	113	18.1 (4.4) [17.2–18.9]	23.7 (4.4) [22.9–24.5]

IHDI, International Hip Dysplasia Institute.

P-value for OAA versus CAA in each IHDI group < 0.01

There were no significant differences between any 2 means for

OAA and CAA, except for the comparison between type I and IV

(p = 0.02), type II and IV (p < 0.001) for OAA; and between type III and IV (p = 0.01), type II and IV (p < 0.001) for CAA.

## Discussion

Although MRI is expensive and sometimes requires sedation, it is still widely used in the examination of immature hips because it does not expose children to radiation and can provide multiplanar, high-quality images that clearly show the boundaries between osseous and cartilaginous structures. A large number of anatomical and MRI studies have shown that MRI can accurately show various morphological changes of the developing hip (Goronzy et al. 2019), but few studies have investigated whether MRI is reliable in measuring the OAA and CAA during development. We found excellent consistency between observers, indicating that the repeatability of MRI measurement is high.

The acetabulum is a ball and socket joint consisting of the anterior pubis, superior ilium, and posterior ischium. In a normal newborn, the acetabulum is a cartilage complex composed of acetabular cartilage and Y-shaped cartilage, and the development of the acetabulum is mainly characterized by endochondral ossification after birth. Li et al. (2016) measured the OAA and CAA of 180 children with normal hips in different age groups from 6 months to 16 years by using MRI, and found that OAA and CAA were both relatively constant among the different age groups. They speculated that this may be due to the compressive stress of the femoral head on the acetabulum being uniform in the normal hip joint, which tends to balance the growth of the ilium, ischia, and pubis, thereby keeping the acetabulum in a stable axial opening direction. Our study results differed from those of Li et al., which may be due to the difference in the age grouping method used or the small sample size of age subgroups in the study by Li et al.

In our cross-section investigation, there was a rapid increase in OAA and CAA in the first 2 years after birth, and stability is maintained from 2 to 9 years of age, showing a similar growth pattern in development of the osseous and cartilaginous acetabular index in children with normal hips as observed by Li et al. (2012). Before adolescence OAA is constant, being approximately 3° less than CAA until 9 years of age, suggesting that ossification in the anterior and posterior walls of the acetabulum has an identical velocity to the development of the cartilaginous acetabular wall. The second developmental stage of OAA is between ages 9 and 16 years, with an increase of 2°–3°. This change is mainly attributed to the fact that ossification of the acetabular posterior wall is faster than that of the anterior wall. Albers et al. (2017) suggested that the OAA increase is related to the appearance of the secondary ossification center in the anterior and posterior walls of the acetabulum that begins at 9 years of age.

Unlike OAA, CAA is almost constant at the level of 15° (SD 3.4) throughout the childhood and adolescence period. This indicates that the cartilaginous acetabular anterior and posterior walls are fully formed at birth. This finding is supported by Johnson et al. (1989), who investigated the anatomical structure of the hip in infants by MRI. However, the postnatal development of cartilaginous acetabular anterior and posterior margins has not been reported previously. Our cross-sectional study was age categorized, with detailed mean and standard deviation from the age of 1 to 16 years, and provides the normal standards for the development of cartilaginous acetabular anterior and posterior walls in children. After infancy, a hip with a CAA exceeding 21° (> 2 SDs from the mean) should be considered as cartilaginous over-anteversion. According to our findings, although OAA and CAA had a significant positive correlation, the anteverted level was different between them. In children during skeletal development, the cartilaginous acetabular edges determine the true AA. Therefore, CAA cannot be replaced by OAA.

In addition, we found that before the age of 9 years, OAA development in boys and girls is basically the same, whereas at the age of 9–16 years, OAA in girls is larger than that of boys. The development of CAA between sexes showed a similar difference to that of OAA. Previous studies also support this conclusion (Rubalcava et al. 2012, Jiang et al. 2015b). However, the reasons for this phenomenon are not clear; the authors speculate that it may be related to the differences in pelvic morphology between men and women.

Nevertheless, there was also a statistically significant correlation between OAA and CAA in the DDH cases, indicating that both OAA and CAA can reflect the degree of excessive anteversion of the acetabulum. However, due to the large individual differences in acetabulum pathology, especially the difference in cartilage acetabulum edge, it was unreasonable to evaluate CAA using OAA directly. We believe that cartilaginous anteversion of the acetabulum is more representative of the final state of acetabulum development than osseous anteversion of the acetabulum (Duffy et al. 2002, Zamzam et al. 2008). It has been reported that the rotational degree of the acetabulum in Salter pelvic osteotomy can be determined according to the degree of AA shown in 3D CT; thus, it may be cursory (Jiang et al. 2015a). We found that the true degree of AA is determined by the degree of CAA, which is often larger than the degree of OAA and should be taken into account in the intraoperative estimation of pelvic rotation. Excessive acetabular anteversion or retroversion can lead to excessive containment, and then lead to impingement between the femoral head and acetabulum, which is an important cause of osteoarthritis (Kim et al. 1999). Owing to the limitation in using intraoperative MRI, it is difficult to evaluate the anteversion of the acetabulum. We suggest that the difference between OAA and CAA should be evaluated preoperatively by MRI, and the true AA (CAA) can be calculated by measuring the OAA using radiological methods. Additionally, one can directly observe the position of the anterior and posterior acetabular edges by intraoperative arthrography according to Forlin’s method (Forlin et al. 1992) to accurately evaluate the anteversion degree of the cartilaginous acetabular edge.

Mootha et al. (2010) used MRI to assess acetabular and femoral anteversion in 45 children with DDH in children aged 12 to 48 months and found that acetabular anteversion was increased in the dislocated group compared with the normal group. In this study, both OAA and CAA demonstrated an excessive anteversion in DDH cases, which is similar to results reported in previous studies (Jacobsen et al. 2006, Kobayashi et al. 2010). Moreover, no difference or only a little variation (approximately 2°–3°) for OAA and CAA was found among the different types of IHDI, indicating that the developmental defect of the anterior margin was a common physiological phenomenon after dislocation, which might be related to the loss of mutual stimulation between the femoral head and the acetabulum. Understanding these physiological characteristics is helpful to guide evaluation of the pathological morphology and surgical correction of DDH.

For uninvolved hips in unilateral DDH cases, both OAA and CAA were increased compared with those of the normal hips when both groups were age matched. It is suggested that both the osseous and cartilaginous acetabulum in the uninvolved hip also have mild over-anteversion; thus, one should be discreet when using the unaffected hip of unilateral DDH as a control. This phenomenon has a similar performance in the observation of other DDH parameters (Li et al. 2012). This is also supported by the study of Song et al. (2008), who suggested that 40% of uninvolved hips in unilateral DDH patients have mild dysplasia.

### Limitations

There are limitations in our study. Although patients with a non-hip disorder were included, they were not healthy children and had diseases pertaining to lower limb pain, benign abdominal tumors, lymphangioma, or hemangioma in the cutaneous or subcutaneous tissues around the pelvis. In addition, with the increasing degree of dislocation in DDH patients, it could be difficult to locate the center of the acetabulum accurately, which may influence the results of measurements.

## Conclusion

The growth patterns of the osseous and cartilaginous parts in the acetabulum are different as measured by MRI. The CAA is fully formed after birth and does not change substantially with age until adulthood. However, the OAA increases rapidly within the first 2 years postnatally, and then remains unchanged until 9 years of age; then, from 9 to 16 years of age, it increases slightly by approximately 2°. Both the osseous and cartilage acetabulum of children with DDH have excessive anteversion. The uninvolved hips of unilateral DDH cases are also over-anteverted compared with normal hips. CAA represents the true AA. Preoperative evaluation of CAA in DDH patients by MRI is of importance for accurate correction of acetabular orientation.
